# Serum Metabolomic Profiling Reveals Potential Mechanisms and Biomarkers of Seladelpar Treatment in Patients With Primary Biliary Cholangitis

**DOI:** 10.1155/ppar/2362528

**Published:** 2026-09-25

**Authors:** Yun-Jung Choi, Andrew Schwab, James Lim, Samantha M. Carlisle, Charles A. McWherter, Jeffrey D. Johnson

**Affiliations:** ^1^ CymaBay Therapeutics, Fremont, California, USA; ^2^ Metabolon Inc., Morrisville, North Carolina, USA; ^3^ Monoceros Biosystems, San Diego, California, USA

**Keywords:** metabolomics, peroxisome proliferator-activated receptor delta, primary biliary cholangitis, seladelpar

## Abstract

**Background:**

Seladelpar, a potent and selective peroxisome proliferator‐activated receptor‐*δ* (PPAR*δ*) agonist, is an approved second‐line therapy for primary biliary cholangitis (PBC) in patients with an inadequate response or intolerance to ursodeoxycholic acid (UDCA). In clinical studies, seladelpar significantly improved biochemical markers associated with disease progression and pruritus. Our objective was to explore the metabolic mechanisms underlying seladelpar′s effects and identify potential biomarkers.

**Methods:**

Untargeted serum metabolomic analyses were conducted using UHPLC‐MS/MS on fasting samples from 160 patients with PBC, collected before and after 12 weeks of treatment with placebo (*n* = 55), seladelpar 5 mg (*n* = 52), or seladelpar 10 mg (*n* = 53) in a randomized, placebo‐controlled Phase 3 study (NCT03602560).

**Results:**

A total of 1474 metabolites were identified from global untargeted serum metabolomics analysis. Random forest, weighted correlation network analysis, and differential abundance analysis revealed significant metabolomic changes after seladelpar treatment. Notable changes included significant increases in carnitine, acetylcarnitine, other acylcarnitines, and branched‐chain amino acid catabolites as well as significant decreases in dicarboxylates, saturated and unsaturated fatty acids, and lipid‐derived inflammatory mediators including ceramides. Targeted metabolomics confirmed seladelpar′s impact on carnitine and acylcarnitines, which facilitate fatty acid *β*‐oxidation. Gene expression analysis of primary human hepatocytes and mouse tissues showed upregulation of carnitine transporter and mitochondrial carnitine shuttle genes with seladelpar treatment.

**Conclusions:**

Seladelpar treatment of PBC patients resulted in broad changes in serum metabolites, many of which are downstream of known PPAR*δ* activation effects, including peroxisomal and mitochondrial fatty acid *β*‐oxidation. This comprehensive metabolomics analysis highlights potential pathways for future exploration in the treatment of PBC and its symptoms.

**Trial Registration:** ClinicalTrials.gov identifier: NCT03602560; EudraCT number: 2018‐001171‐20

## 1. Introduction

Primary biliary cholangitis (PBC) is a chronic, inflammatory, autoimmune liver disease characterized by progressive destruction of the intrahepatic bile ducts [1, 2]. PBC is a rare disease disproportionately impacting middle‐aged women. PBC is often asymptomatic at early stages and is usually diagnosed by positive antimitochondrial antibodies (AMA) with an accompanying elevated level of alkaline phosphatase (ALP) and other liver markers. The etiology and pathogenesis of PBC are not well understood, and this limits the development of effective therapies. There is no cure for PBC, and currently available first‐line treatment with ursodeoxycholic acid (UDCA) is not always sufficient to prevent disease progression from fibrosis to cirrhosis and, eventually, liver failure. Recently approved peroxisome proliferator‐activated receptor (PPAR) agonists are promising, and in clinical studies, they demonstrated improvement in cholestatic markers and, in the case of seladelpar, significant improvement in pruritus [3–5].

Seladelpar is a potent and selective agonist of peroxisome proliferator‐activated receptor‐*δ* (PPAR*δ*), a nuclear receptor transcription factor that regulates a wide range of metabolic pathways [6, 7]. In clinical studies, seladelpar treatment improved the biochemical markers of cholestasis associated with disease progression in PBC and significantly improved pruritus in patients with moderate to severe pruritus at baseline [3, 4, 8, 9].

Serum metabolomics represents a powerful and noninvasive tool to detect biochemical changes altered by the progression of disease or with therapeutic response. Simultaneous analysis of biochemicals in the serum at the time of sample collection provides a comprehensive profile of metabolic status. Utilizing metabolomic information in clinical studies for treatment‐induced changes enhances our understanding of mechanisms of drug efficacy and is likely to be crucial for biomarker discovery.

Serum metabolomic analysis in patients with PBC compared to healthy subjects revealed a distinct metabolic profile and metabolic pathways contributing to the pathogenesis of this still poorly understood disease. With the progression of PBC, increased levels of bile acids were observed, whereas the levels of carnitine and short‐chain acylcarnitines (propionyl carnitine and butyryl carnitine) were decreased [10]. These findings were consistent with a serum metabolomic study with PBC patients that revealed an elevation of conjugated primary bile acids, ketone bodies, free fatty acids, long‐chain saturated/unsaturated fatty acids, and medium‐/long‐chain acylcarnitines, suggestive of decreased fatty acid oxidation in patients with PBC [11].

Cholestasis is associated with impaired mitochondrial function, resulting in fuel switching from fatty acid *β*‐oxidation toward compensatory glycolysis [12]. Systemic mitochondrial dysfunction was reported in a detailed metabolic study of PBC patients after exercise [13]. There was no correlation between the phosphocreatine and adenosine diphosphate recovery rates in PBC patients, contrasting with the close correlation between these two rates (normal mitochondrial function) found in normal, primary sclerosing cholangitis (PSC), or chronic fatigue patients. PBC patients had marked decreases in muscle pH (excess acidosis) during exercise and delayed recovery of pH to the resting state value after exercise.

In this study, we used untargeted global serum metabolomics to gain mechanistic insight into metabolic pathways that were altered by seladelpar treatment to explain the improvement in biochemical markers seen in patients with PBC in our clinical studies. Seladelpar broadly lowered serum free fatty acids, long‐chain saturated and unsaturated fatty acids, and ceramides and raised the levels of carnitine and short‐, medium‐, and long‐chain acylcarnitines as well as catabolites of branched‐chain amino acids. These observations provide insight into the mechanisms by which seladelpar improves and potentially expands the repertoire of biochemical markers of PBC.

## 2. Methods

### 2.1. Study Design, Patients, and Treatment

The ENHANCE study was a Phase 3, randomized, double‐blind, placebo‐controlled study of seladelpar in patients with PBC; details of the study design have been reported previously [ClinicalTrials.gov (NCT03602560) and EU Clinical Trials Registry (EudraCT2018‐001171‐20)] [3]. The ENHANCE study protocol was approved by appropriate local and national institutional review boards or independent ethics committees, and the trial was conducted in accordance with the Declaration of Helsinki and Good Clinical Practice guidelines. All patients provided written informed consent.

Patients aged 18–75 years, diagnosed with PBC, and either showing an incomplete response or intolerance of UDCA were recruited at 111 sites in 21 countries. Eligible patients had an ALP level greater than 1.67 times the upper limit of the normal range (ULN) despite stable doses of UDCA for the past 1 year, if tolerated, and a total bilirubin level of less than two times the ULN. Patients were randomly assigned in a 1:1:1 ratio to receive once‐daily oral placebo, seladelpar 5 mg, or seladelpar 10 mg. In this report, the serum metabolome of patients was examined using fasting serum samples collected on Day 1 and after 12 weeks of treatment with placebo, seladelpar 5 mg, or seladelpar 10 mg. The ethics committee approved the protocol, and informed consent permitted analyzing collected samples for potential biomarkers.

### 2.2. Untargeted Analysis of Serum Metabolomics

Untargeted metabolomic analysis was performed at Metabolon Inc. (Morrisville, North Carolina, United States). Samples were extracted with methanol to precipitate protein and dissociate small molecules bound to protein or trapped in the precipitated protein matrix. The resulting extract containing chemically diverse metabolites was dried then reconstituted in solvents compatible to each of the four methods: two for analysis by two separate reverse phase (RP)/Ultrahigh Performance Liquid Chromatography‐Tandem Mass Spectroscopy (UPLC‐MS/MS) methods using positive ion mode electrospray ionization (ESI), one for analysis by RP/UPLC‐MS/MS using negative ion mode ESI, and one for analysis by HILIC/UPLC‐MS/MS using negative ion mode ESI. All methods utilize a Waters ACQUITY UPLC and a Thermo Scientific Q‐Exactive high‐resolution/accurate mass spectrometer interfaced with a heated electrospray ionization (HESI‐II) source and Orbitrap mass analyzer operated at 35,000 mass resolution. The scan range varied slightly between methods but covered approximately 70–1000 m/z. Metabolites were identified by comparison to a referenced library of chemical standards. Metabolite levels were quantified by area‐under‐the‐curve analysis for peak area. To ensure high quality of the dataset, control and curation processes were subsequently used to ensure true chemical assignment and remove artifacts and background noise.

### 2.3. Targeted Metabolomics Analysis of Carnitine and Acylcarnitines

Serum samples were analyzed for carnitine and 17 acylcarnitines by LC‐MS/MS: carnitine, acetyl carnitine (C2:0), propionyl carnitine (C3:0), butyryl carnitine (C4:0), isobutyryl carnitine (C5:0), valeryl carnitine (C6:0), isovaleryl carnitine (C5:0), 2‐methylbutyryl carnitine (C4:0), 3‐hydroxybutyryl carnitine (C4:0), hexanoyl carnitine (C6:0), octanoyl carnitine (C8:0), decanoyl carnitine (C10:0), dodecanoyl carnitine (C12:0), myristoyl carnitine (C14:0), palmitoyl carnitine (C16:0), linoleoyl carnitine (C18:2), oleoyl carnitine (C18:1), and stearoyl carnitine (C18:0). Calibration samples were prepared at 10 different concentration levels by spiking phosphate buffered saline with corresponding calibration spiking solutions. Calibration samples, study samples, and quality control samples were then spiked with a solution of isotopically labeled internal standards and subjected to protein precipitation with an organic solvent (acidified methanol). Following centrifugation, an aliquot was injected onto an Agilent 1290/SCIEX QTRAP 5500 LC‐MS/MS system equipped with a C18 reversed phase UHPLC column. The mass spectrometer was operated in positive mode ESI. The peak area of the individual analyte product ions was measured against the peak area of the product ions of the corresponding internal standards. Quantitation was performed using a weighted linear least squares regression analysis generated from fortified calibration standards prepared concurrently with study samples.

### 2.4. Gene Expression of Carnitine Transporter and Mitochondrial Shuttle Genes

Cryopreserved primary human hepatocytes were obtained from LifeNet Health LifeSciences and thawed in Hepatocyte Thaw Medium (Thermo Fisher Scientific, Cat# CM7500) and cultured according to the manufacturer′s instructions. Authorization was obtained from the donor, or the donor′s legal next of kin, for use of the tissue and its derivatives for research purposes. The mouse tissues used to measure the gene expression were from the study published earlier [7]. At the end of the study, mice were terminated by cardiac puncture under isoflurane anesthesia, and tissues were collected. All mouse experiments were approved by The Animal Experimentation Council, Danish Veterinary and Food Administration (License No. 2013‐15‐2934‐00784) and the Gubra ethics committee. Animal experiments were conducted at Gubra (Hørsholm, Denmark) in compliance with internationally accepted principles for the care and use of laboratory animals.

To explore whether seladelpar affects the expression levels of carnitine transporter, carnitine synthesis, and mitochondrial shuttle genes, we measured OCTN2, CPT1A, CPT2, CACT, CRAT, and BBOX1 from primary human hepatocytes incubated with 10‐*μ*M seladelpar for 24 h. Male C57BL/6J mice were orally dosed with vehicle or seladelpar (10 mg/kg/day) for 12 weeks, and RNA‐seq was performed using terminal tissues (intestine, liver, kidney, and skeletal muscle). The same genes were also examined for expression level of the mouse orthologs. PrimeTime qPCR primer assays (Integrated DNA Technologies) for RT‐qPCR analysis were used. Gene expression levels were calculated using the *ΔΔ*Ct method.

### 2.5. Statistical Analysis

For each metabolite, raw values in experimental samples were normalized by dividing by the median value of the corresponding instrument batch to mitigate interexperiment effects. Missing values, labeled to be below the limits of detection, were imputed with the observed minimum value for each metabolite.

Matched random forest analyses were performed to identify distinguishing features between Day 1 and Week 12 by treatment group using the randomForest package in R (Version 4.3.3). The inputs for random forests classifying placebo Day 1 versus Week 12, seladelpar 10 mg Day 1 versus Week 12, and seladelpar 5 mg Day 1 versus Week 12 utilized 110 (55 subjects), 106 (52 subjects), and 106 (53 subjects) samples, respectively, and all three random forests analyzed all 1474 features. The matched random forest algorithm result is the collective result of a large number of random forests, one random forest per subject. The algorithm leaves out one subject, a random forest is run, and the left‐out subject is predicted. Each individual random forest was configured with 50,000 trees (ntree = 50,000) and 38 (the default mtry setting equal to the sqrt of the number of features) randomly selected predictors at each split. Fifty thousand trees allowed for the convergence of the Out‐of‐Bag (OOB) error rate. Feature importance was assessed using the mean decrease in accuracy. For each comparison, the mean decreases in accuracies were averaged across the collection of random forests. The model performance was evaluated using the OOB error rate.

Differentially abundant metabolites were identified using the limma package in R with the limma‐trend method, which applies linear modeling and empirical Bayes techniques. Given the paired nature of the samples, within‐patient correlations were accounted for using duplicate correlation analysis. Data were imputed, normalized, and log_2_‐transformed prior to analysis. A design matrix was constructed to represent experimental conditions and sample pairing, and the correlation estimate was incorporated into the linear model. Moderated *t* statistics, adjusted *p* values, and log‐fold changes were calculated. Metabolites with adjusted *p* values (false discovery rate [FDR] < 0.05) based on the Benjamini–Hochberg method were considered differentially abundant. This approach ensured accurate analysis of paired samples while appropriately accounting for within‐patient correlations.

Weighted correlation network analysis (WCNA) was conducted using the WGCNA package (Version 1.72‐5) on model‐adjusted metabolite abundance data from paired patient samples. An unsigned network was constructed, and a soft‐thresholding power of 28 was selected to approximate scale‐free topology, determined by the point where the fit to this topology plateaued. Using this threshold, a topological overlap matrix (TOM) was generated, and metabolites were hierarchically clustered into modules with a minimum module size of 30 metabolites. Module eigen‐metabolites, calculated as the first principal component of each module, were compared across treatment groups to identify modules reflecting treatment effects independently of placebo. Hub metabolites, defined as those with the highest intramodular connectivity, were prioritized for further analysis as potential key drivers of metabolic changes in PBC patients.

## 3. Results

### 3.1. Patient Demographics and Serum Biochemistry

A total of 160 patients with PBC comprising all patients having evaluable serum samples from both Day 1 and Week 12 were included in the serum metabolomics analysis. The patients had been randomly assigned to one of three treatment groups: placebo (*n* = 55), seladelpar 5 mg (*n* = 52), or seladelpar 10 mg (*n* = 53). Demographics and pre‐ and post‐treatment biochemistry are summarized in Table 1. Demographics and serum biochemistry were well balanced among treatment groups. The majority of PBC patients were female (94%), with a mean age of 56 years and a body mass index (BMI) of 29 kg/m^2^. They had elevated ALP levels (mean 274 U/L), 90% were AMA‐positive, and 95% were on UDCA.

**Table 1:** Demographics and serum biochemistry of PBC patients at Day 1 and Week 12.

Demographics	Placebo		Seladelpar 5 mg		Seladelpar 10 mg	
	(*n* = 55)		(*n* = 52)		(*n* = 53)	
Female, *n* (%)	54 (98%)		47 (90%)		50 (94%)	
Age, years	56 (7)		56 (9)		57 (10)	
BMI, kg/m^2^	29 (6)		28 (5)		28 (7)	
White, *n* (%)	49 (89%)		49 (94%)		46 (87%)	
AMA‐positive, *n* (%)	49 (89%)		48 (92%)		47 (89%)	
UDCA received, *n* (%)	54 (98%)		49 (94%)		49 (92%)	
Biochemistry	Day 1	Week 12	Day 1	Week 12	Day 1	Week 12
ALP (U/L)	282 (105)	281 (116)	282 (126)	179 (56)	263 (96)	147 (56)
ALT (U/L)	41 (20)	43 (23)	47 (24)	35 (18)	42 (20)	38 (26)
AST (U/L)	35 (14)	36 (16)	39 (18)	35 (16)	38 (14)	38 (19)
GGT (U/L)	200 (153)	203 (177)	204 (163)	141 (112)	208 (154)	134 (111)
Total bilirubin (mg/dL)	0.7 (0.3)	0.7 (0.3)	0.7 (0.3)	0.7 (0.3)	0.6 (0.3)	0.6 (0.3)
Total cholesterol (mg/dL)	233 (53)	231 (53)	215 (45)	209 (45)	231 (56)	221 (55)
LDL‐cholesterol (mg/dL)	137 (42)	136 (42)	120 (36)	113 (33)	129 (43)	120 (45)
HDL‐cholesterol (mg/dL)	73 (22)	72 (23)	75 (22)	77 (25)	75 (22)	80 (22)
Triglycerides (mg/dL)	117 (55)	116 (44)	101 (48)	96 (43)	125 (77)	101 (55)
Albumin (g/dL)	4.2 (0.2)	4.2 (0.3)	4.1 (0.3)	4.2 (0.3)	4.1 (0.3)	4.2 (0.3)
Total protein (g/dL)	7.6 (0.6)	7.5 (0.6)	7.4 (0.4)	7.4 (0.4)	7.5 (0.6)	7.6 (0.6)
Urea (mg/dL)	15.4 (3.9)	14.7 (3.8)	15.5 (4.6)	15.8 (4.0)	14.9 (4.9)	15.7 (4.5)
Creatinine (mg/dL)	0.71 (0.13)	0.72 (0.13)	0.72 (0.18)	0.75 (0.16)	0.76 (0.23)	0.79 (0.16)

*Note:* Unless otherwise stated, all data are shown as mean (SD).

Abbreviations: ALP, alkaline phosphatase; ALT, alanine aminotransferase; AMA, anti‐mitochondrial antibody; AST, aspartate aminotransferase; BMI, body mass index; GGT, gamma‐glutamyl transferase; HDL, high‐density lipoprotein; LDL, low‐density lipoprotein; PBC, primary biliary cholangitis; UDCA, ursodeoxycholic acid.

### 3.2. Differential Abundance

**Figure 1:**
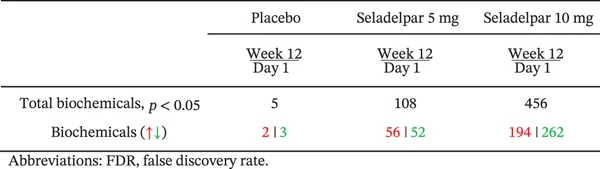
Significant changes in differentially abundant metabolites were observed after 12 weeks of treatment (FDR < 0.05). The direction of these changes is indicated in red (increase) or green (decrease). Abbreviation: FDR, false discovery rate.

**Figure 2:**
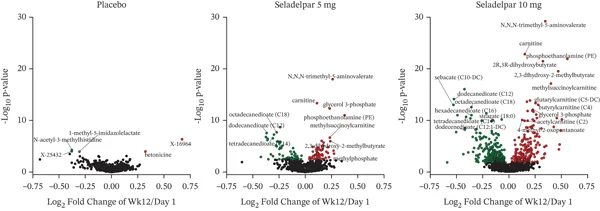
Volcano plots exhibit the significance of changes in serum metabolites following seladelpar treatment. The *x*‐axis represents the mean fold change (Week 12/Day 1), whereas the *y*‐axis shows the significance (*p* value) of differential metabolites. Red and green dots indicate metabolites with a significant increase or decrease at Wk 12 compared to Day 1 (FDR < 0.05), respectively. Black dots represent metabolites with no significant change (FDR  ≥  0.05). Abbreviations: FDR, false discovery rate; Wk, week.

A total of 1474 metabolites (1171 named and 303 unnamed) were identified in the global untargeted analysis of human serum from patients with PBC. Metabolite abundances were compared between Day 1 and Week 12 samples within each treatment group (placebo, seladelpar 5 mg, and seladelpar 10 mg), using Patient ID as a random effect to account for paired samples. After 12 weeks of treatment, five metabolites showed significant changes in the placebo group, 108 metabolites in the seladelpar 5 mg group, and 456 metabolites in the seladelpar 10 mg group (FDR < 0.05; Figure 1). Of the 108 metabolites that were significantly changed in the seladelpar 5 mg group, 92 were also significant in the seladelpar 10 mg group. Of these, 75 exhibited a greater fold change in the seladelpar 10 mg group, indicating dose‐dependency in metabolite levels. Robust metabolomic shifts were observed in the seladelpar treatment groups, as visualized by volcano plots (Figure 2).

The most common and significantly increased metabolites following seladelpar treatment were carnitine, acylcarnitines, and branched‐chain amino acid catabolites. In contrast, seladelpar treatment significantly decreased the levels of dicarboxylates, fatty acids, and ceramides.

### 3.3. Random Forest Analysis

**Figure 3:**
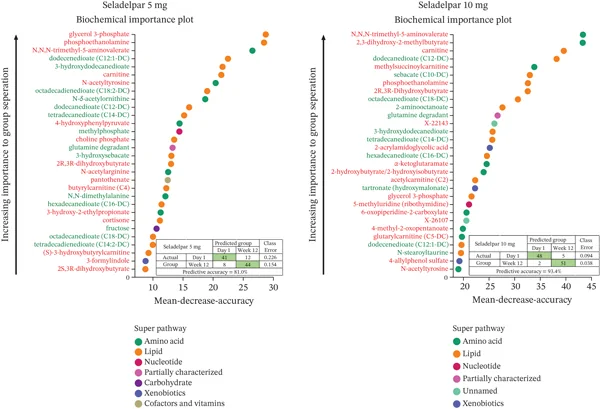
Random forest analysis was performed on metabolites after 12 weeks of treatment with seladelpar 5 or 10 mg. Red letters indicate an increase in metabolite levels at Week 12 compared to Day 1, whereas green letters indicate a decrease.

Random forest analysis was used to identify metabolites that differentiated Day 1 and Week 12 samples following treatment with seladelpar (Figure 3). The random forest model showed predictive accuracy of 81% for the 5 mg and 93.4% for the 10 mg seladelpar groups. Among the top 30 metabolites ranked by their importance for the effect of seladelpar 10 mg treatment, the most common included dicarboxylates, carnitines (both free carnitine and acylcarnitines), and branch‐chain amino acid metabolites. The metabolite that most differentiated the seladelpar 10 mg–treated serum samples was *N,N,N*‐trimethyl‐5‐aminovalerate (TMAVA or 5‐aminovaleric acid betaine), followed by 2,3‐dihydroxy‐2‐methylbutyrate, carnitine, and dodecanedioate (C12‐DC). Random forest classification also identified 40% of metabolites in common between the 5 and 10 mg seladelpar treatments.

### 3.4. WCNA

**Figure 4:**
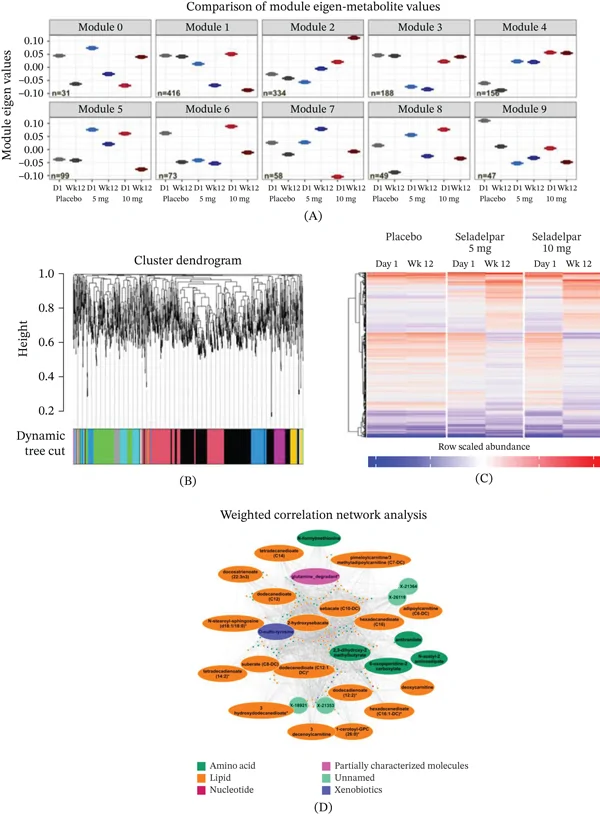
WCNA identifies co‐abundant metabolite modules distinguishing baseline and seladelpar‐treated samples. (A) Comparison of 10 module eigen‐metabolite values identifies Module 1 as a treatment‐specific module. (B) The cluster dendrogram shows the hierarchical clustering of metabolites, with branches representing metabolite modules identified through WCNA. Each module is color‐coded. (C) The heat map of Module 1 metabolites highlights treatment‐specific changes in metabolite abundance. Rows represent individual metabolites (scaled abundance values), and columns represent treatment groups (placebo, seladelpar 5 mg, and seladelpar 10 mg) and time point (D1, Wk12). (D) The network visualization of Module 1 displays hub metabolites (larger, labeled nodes) and their connections within the module. Metabolites are color‐coded by their super pathway, including lipids, amino acids, carbohydrates, and others. Abbreviations: D, day; WCNA, weighted correlation network analysis; Wk, week.

WCNA was used to identify metabolite modules that are co‐abundant between baseline and treated samples. By analyzing correlations among metabolites across samples, WCNA revealed metabolic patterns that were not apparent through single‐metabolite or differential abundance analyses. Ten distinct metabolite modules were identified, each representing groups of highly interconnected metabolites (Figure 4A,B). Module 1, which consists of 416 metabolites, showed clear differences between baseline, placebo, and seladelpar‐treated groups, as indicated by the module′s eigen‐metabolite values (Figure 4A). The heat map for Module 1 highlights treatment‐specific changes in metabolite abundances, many of which displayed a dose‐dependent response (Figure 4C). Hub metabolites within this module, shown in Figure 4D, serve as key descriptors of seladelpar treatment. Additionally, the use of unsigned WCNA, which includes both positively and negatively correlated metabolites, provided deeper insights into how seladelpar treatment impacts diverse metabolic pathways, including those involving dicarboxylates, acylcarnitines, ceramides, branch‐chain amino acid catabolites, lipids, gamma‐glutamyl amino acids, and unnamed metabolites.

### 3.5. Major Classes of Metabolites Affected by Seladelpar Treatment

**Figure 5:**
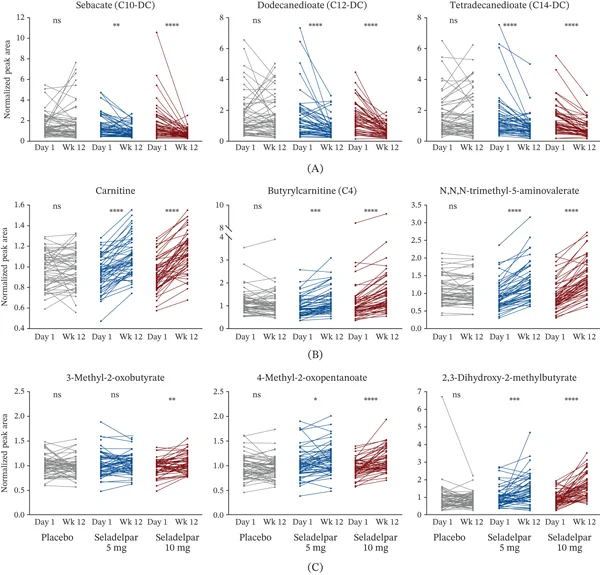
Seladelpar treatments resulted in substantial changes in (A) dicarboxylates, (B) carnitine, butyryl carnitine (C4), *N,N,N*‐trimethyl‐5‐aminovalerate, and (C) branched‐chain amino acid catabolites levels from Day 1 to Wk12 in individual PBC patients. Statistical significance was determined by the Benjamini–Hochberg method (ns: FDR ≥ 0.05,  ^∗^FDR < 0.05,  ^∗∗^FDR < 0.01,  ^∗∗∗^FDR < 0.001, and  ^∗∗∗∗^FDR < 0.0001). Abbreviations: FDR, false discovery rate; Wk, week.

Differential abundance analysis, random forest, and WCNA identified an overlapping set of key metabolites affected by seladelpar treatment, leading a broad range of changes in the abundance of several metabolite classes, including dicarboxylates, carnitines, branched‐chain amino acid catabolites, fatty acids, ceramides, and others. Representative examples of changes in dicarboxylates, carnitines, carnitine‐related metabolites, and branched‐chain amino acid catabolites in individual PBC patients are shown in Figure 5.

#### 3.5.1. Dicarboxylates

**Figure 6:**
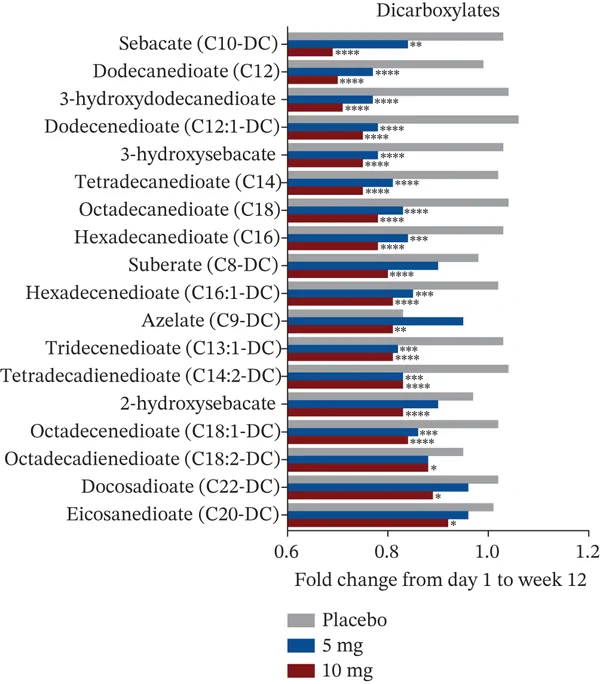
Fold changes of dicarboxylate species with varying chain lengths detected by differential abundance from Day 1 to Week 12. Statistical significance was determined by the Benjamini–Hochberg method (ns: FDR  ≥ 0.05,  ^∗^FDR < 0.05,  ^∗∗^FDR < 0.01,  ^∗∗∗^FDR < 0.001, and  ^∗∗∗∗^FDR < 0.0001). Abbreviation: FDR, false discovery rate.

The most notable changes following seladelpar treatment detected by differential abundance, random forest, and WCNA in this study were in dicarboxylates. In random forest analysis, six of the top 30 metabolites in the seladelpar 10 mg group were dicarboxylate species, including dodecanedioate (C12‐DC), sebacate (C10‐DC), octadecanedioate (C18‐DC), tetradecanedioate (C14‐DC), hexadecanedioate (C16‐DC), and dodecenedioate (C12:1‐DC). WCNA also identified 12 out of 29 hub metabolites as dicarboxylates (Figure 4D). Dicarboxylates are derived from diet or *ω*‐oxidation of free fatty acids. Among the 18 dicarboxylate species with varying chain lengths detected by differential abundance, seladelpar 10 mg significantly decreased all 18, and 15 dicarboxylates were significantly decreased by seladelpar 5 mg treatment, whereas none were significantly changed by placebo treatment (Figure 6). Changes in sebacate (C10‐DC), dodecanedioate (C12‐DC), and tetradecanedioate (C14‐DC) in individual PBC patients are shown in Figure 5A.

#### 3.5.2. Carnitines

Carnitine and acylcarnitines play key roles in facilitating fatty acid *β*‐oxidation in mitochondria and peroxisomes, helping to remove excess and potentially toxic lipid species that result from mitochondrial dysfunction in PBC patients. In the random forest and WCNA of seladelpar treatment, carnitines were the second most commonly identified metabolites. Among the 62 carnitine species detected, 30 showed a significant increase after treatment with 10 mg of seladelpar (as shown by differential abundance) with a clear dose‐dependent response, unlike the placebo group, where no changes were observed. Targeted measurements of carnitine and acylcarnitines confirmed the findings (Figure 7A) of the untargeted analysis (Figure 5B). Seladelpar treatment led to a dose‐dependent increase in both carnitine and shorter‐chain acylcarnitines, with carnitine and acetylcarnitine being the most abundant species (Figure 7A,B). After 12 weeks of seladelpar treatment, carnitine and acetylcarnitine levels increased significantly by 36% (*p* < 0.0001) compared to Day 1.

Figure 7Targeted metabolomics of carnitine and acylcarnitines: Seladelpar treatment resulted in a dose‐dependent increase in carnitine and shorter chain acylcarnitines via upregulation of plasma membrane carnitine transporter OCTN2 and mitochondrial shuttle genes. (A) Carnitine and acylcarnitine levels at Wk12. An inset heat map illustrates the percent change in carnitine and acylcarnitine levels from Day 1. (B) Changes in carnitine, acetyl carnitine, and 3‐hydroxybutyryl carnitine levels in individual PBC patients. (C) Human primary hepatocytes were incubated with 10‐*μ*M seladelpar for 24 h, and qPCR was performed. Data are presented as fold change over vehicle. (D) Male C57BL/6J mice were orally dosed with vehicle or seladelpar (10 mg/kg/day) for 12 weeks, and RNA‐seq was performed using terminal tissues (intestine, liver, kidney, and skeletal muscle). RNA‐seq data are presented as Reads Per Kilobase per Million mapped reads (RPKM). Data are presented as mean ± SE. Statistical significance was determined by a paired or unpaired Student′s *t* test or one‐way ANOVA, where appropriate. Statistical significance is indicated as ns, not significant;  ^∗^ *p* < 0.05,  ^∗∗^ *p* < 0.01,  ^∗∗∗^ *p* < 0.001, and  ^∗∗∗∗^ *p* < 0.0001. Abbreviations: PBC, primary biliary cholangitis; Wk, week.

**Figure:**
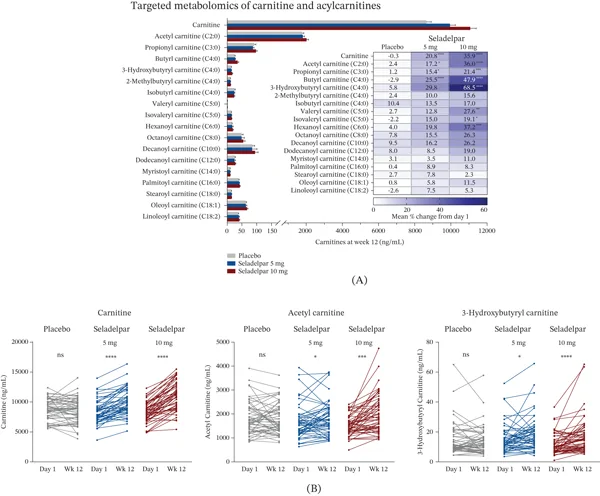

**Figure:**
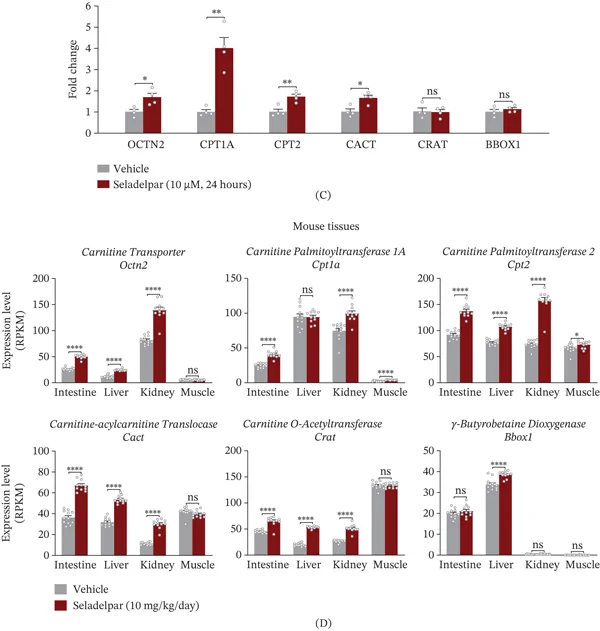

Carnitine homeostasis is primarily maintained through dietary absorption, and by a modest biosynthesis rate from lysine and methionine, and by efficient renal reabsorption [14]. The metabolite TMAVA was identified as the most important differentiating metabolite by random forest analysis in the seladelpar 10 mg group, showing a dose‐dependent increase. This metabolite is structurally similar to carnitine and serves as a substrate for the plasma membrane carnitine transporter OCTN2 (SLC22A5), which is ubiquitously present in tissues [15]. OCTN2 plays a critical role in the absorption of carnitine from diet and its distribution to tissues for fatty acid *β*‐oxidation [16]. Based on these findings, we investigated OCTN2 as a target gene for seladelpar treatment. In primary human hepatocytes, seladelpar increased the expression of *OCTN2* and genes involved in mitochondrial carnitine shuttle (*CPT1A*, *CPT2*, *CACT*, and *CRAT*), whereas no change was observed in *γ*‐butyrobetaine dioxygenase (BBOX1), the rate‐limiting enzyme of carnitine biosynthesis (Figure 7C). In a mouse model of diet‐induced obesity, seladelpar upregulated expression of *Octn2* and mitochondrial shuttle genes in the intestine, liver, and kidney (Figure 7D).

#### 3.5.3. Branched‐Chain Amino Acids

The branched‐chain amino acids (leucine, isoleucine, and valine) are essential constituents of proteins and serve as crucial energy sources [17]. Branched‐chain amino acids are first catabolized by branched‐chain aminotransferase (BCAT) to their *α*‐keto acid intermediates, which are then further catabolized by mitochondrial branch‐chain *α*‐keto acid dehydrogenase (BCKDH) complex. This process leads to either anabolic pathways (such as gluconeogenesis or fatty acid synthesis) or energy generation pathways (e.g., the tricarboxylic acid cycle). While the branched‐chain amino acids themselves did not show significant changes, there were significant and dose‐dependent increases in their catabolites after seladelpar treatment. The branched‐chain amino acid catabolite 2,3‐dyhydroxy‐2‐methylbutyrate was identified as the second most important metabolite by random forest analysis and a key hub metabolite by WCNA of seladelpar 10 mg group. Other branched‐chain amino acid catabolites, including 3‐methyl‐2‐oxobutyrate, 3‐methyl‐2‐oxovalerate, and 4‐methyl‐2‐oxopentanoate, also showed increased differential abundance after 12 weeks of seladelpar 10 mg treatment (Figure 5C).

#### 3.5.4. Fatty Acids

Treatment with seladelpar 10 mg for 12 weeks resulted in significant decreases in all the saturated fatty acids detected (C10, C12, C14, C15, C16, C17, C18, C19, and C20) and many unsaturated fatty acid species. Seladelpar 5 mg treatment showed a trend toward decreasing both saturated and unsaturated fatty acids, but these changes did not reach statistical significance. No significant changes in fatty acid levels were observed in the placebo group.

#### 3.5.5. Ceramides

Ceramides have been shown to have pathological consequences when present at elevated levels in serum. As found for the dicarboxylates and fatty acids, seven out of 10 detected ceramides were significantly reduced by seladelpar 10 mg treatment. These ceramides include *N-*stearoyl‐sphinganine (d18:0/18:0), ceramide (d18:1/17:0, d17:1/18:0), ceramide (d18:1/14:0, d16:1/16:0), *N-*palmitoyl‐sphinganine (d18:0/16:0), *N-*palmitoyl‐heptadecasphingosine (d17:1/16:0), *N-*stearoyl‐sphingosine (d18:1/18:0), and ceramide (d18:2/24:1, d18:1/24:2). In the seladelpar 5 mg group, there was a trend toward a decrease in ceramide levels, though it was not statistically significant.

#### 3.5.6. Others

Seladelpar treatments led to a decrease in gamma‐glutamyl amino acids, which was consistent with reduction of gamma‐glutamyl transferase activity seen in PBC patients treated with seladelpar. Additionally, seladelpar 10 mg treatment resulted in increased *N*‐acetylamines. Changes were also observed in other lipid groups, such as phospholipids, lysophospholipids, sphingomyelins, and plasmalogens, with some species within each group increasing and others decreasing. Notably, 12‐HETE, an inflammatory lipid mediator derived from arachidonic acid (C20:4), was significantly reduced by seladelpar 10 mg treatment. Plant‐derived sterols such as beta‐sitosterol and campesterol which serve as markers of cholesterol absorption, were significantly decreased by seladelpar 10 mg treatment. This effect of seladelpar was observed in previous clinical studies [18, 19].

Glycerol 3‐phosphate serves as a substrate for triglyceride synthesis and is a key intermediate in glycolysis. It plays a critical role in the glycerol 3‐phosphate shuttle, a pathway that transports electrons produced during glycolysis across the inner mitochondrial membrane for oxidative phosphorylation, by oxidizing cytoplasmic NADH to NAD+ [20]. In the random forest analysis, glycerol 3‐phosphate was identified in both the seladelpar 5 and 10 mg groups, where it was significantly increased by seladelpar treatment.

### 3.6. Significant Correlation of Changes in ALP and Changes in Metabolites

**Figure 8:**
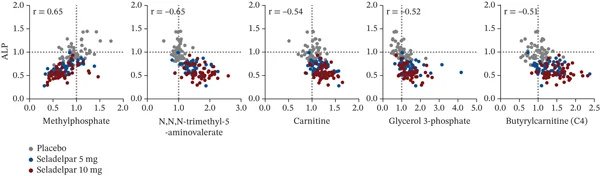
Close correlations between changes in ALP and changes in metabolites, expressed as the Week 12/Day 1 ratio, are shown. Seladelpar treatments produced a dose‐dependent shift away from a ratio of 1. Each dot represents an individual patient. Only correlations with an absolute correlation coefficient >|0.5| (positive or negative) are displayed. Both the *x*‐ and *y*‐axes represent the Week 12/Day 1 ratio. Abbreviation: ALP, alkaline phosphatase.

In this Phase 3 study, treatment with seladelpar 10 mg for 12 weeks resulted in a significant reduction in ALP, an accepted efficacy biomarker of PBC. To identify metabolomic markers associated with treatment response, associations between treatment‐related changes in ALP and changes in metabolites were evaluated using the ratio of Week 12 to Day 1 values for both ALP and metabolites (Figure 8).

In the placebo group, most patients clustered near a ratio of 1 for both ALP change and metabolite change, indicating minimal changes in ALP and metabolite levels over time. In contrast, seladelpar‐treated patients showed dose‐dependent shifts away from 1, suggesting concordant changes in ALP and metabolite levels. Changes in methylphosphate were positively correlated with changes in ALP (*r* = 0.65), whereas changes in TMAVA (*r* = −0.65), carnitine (*r* = −0.54), glycerol‐3‐phosphate (*r* = −0.52), and butyrylcarnitine (*r* = −0.51) were negatively correlated with ALP changes. Although it remains unclear whether these metabolites directly mediate the treatment‐related reduction in ALP, they may serve as potential biomarkers of seladelpar treatment response.

## 4. Discussion

Seladepar (Livdelzi) is an approved second‐line therapy for the treatment of PBC. It is used in combination with UDCA for patients who have an inadequate response to UDCA or as monotherapy in UDCA‐intolerant patients. Seladelpar improves biochemical markers associated with disease progression and alleviates pruritus in patients with PBC, while also being overall safe and well tolerated in clinical trials [4, 18, 21]. Improvements in sleep disturbance and measure of fatigue have also been shown to improve in patients with PBC and pruritus who were treated with seladelpar [22]. Effects observed in the registrational Phase 3 RESPONSE study were sustained with treatment for up to 2 years [23]. The treatment goal for PBC is to prevent or slow the progression of liver fibrosis and reduce debilitating symptoms, such as pruritus and fatigue, commonly experienced by PBC patients.

This untargeted metabolomics study involved a large sample size (*n* > 50 per group) from a randomized, placebo‐controlled clinical trial in PBC patients to explore metabolomic changes after 12 weeks of seladelpar treatment by comparing pre‐ and post‐treatment paired serum samples. The current state‐of‐the‐art LC‐MS/MS analysis of fasting serum samples enabled the detection of over 1400 metabolites, approaching a comprehensive set of mechanistic and diagnostic biomarkers to assess the activity of seladelpar in treating PBC. Analyzing paired samples allowed each patient to serve as their own baseline control, minimizing interindividual variability and providing a robust measure of treatment‐related metabolic changes. This design is especially important because metabolic profiles can vary widely among individuals due to genetics, diet, disease status, medication use, lifestyle, and environmental exposures. Given this inherent variability in metabolite levels, all available on‐treatment samples were included to maximize sample size and strengthen the analysis. Seladelpar is a potent and selective human PPAR*δ* agonist; therefore, the metabolomic changes observed in this study are likely to reflect PPAR*δ*‐specific biological activity. Our large‐scale metabolomics study could serve as a reference for future comparisons of different PPAR agonists currently in clinical use to define the specific biological activities of each agent.

Random forest, WCNA, and differential abundance analyses of the data revealed overlapping patterns of metabolite changes following seladelpar treatment in PBC patients. In this clinical trial, which tested 5 and 10‐mg doses of seladelpar, we found that many of the prominent metabolite changes were dose dependent. At the 10‐mg dose (the currently prescribed dose), 17% of the identified metabolites significantly increased, whereas 22% decreased with seladelpar treatment.

The most prominent class of increased metabolites was carnitines, which we confirmed to be substantially elevated by targeted quantitation. Free carnitine and acetylcarnitine levels were also elevated with 10 mg seladelpar treatment, as was TMAVA, which is also a substrate of the carnitine transporter OCTN2 [15, 16]. Upregulation of OCTN2 by the PPAR*δ* agonist GW0742 resulted in increased carnitine uptake in bovine kidney cells [24]. The consistent and significant increase in carnitine and TMAVA levels may be due to seladelpar′s upregulation of OCTN2. The observed increase in carnitine levels following seladelpar treatment is likely due to enhanced dietary carnitine uptake and its reabsorption through OCTN2 upregulation. Seladelpar′s upregulation of the carnitine transporter expression in the liver, intestine, and kidney may contribute to the increase in serum carnitine levels. Overall, the broad upregulation of OCTN2 by seladelpar may help boost intracellular carnitine levels to support enhanced fatty acid *β*‐oxidation. TMAVA was the most differentiating molecule affected by seladelpar treatment in the random forest and could serve as a promising pharmacodynamic marker for seladelpar. The upregulation of OCTN2 and mitochondrial shuttle genes, along with the resulting increase in carnitine levels, likely contributes to the improved capacity for fatty acid *β*‐oxidation by seladelpar treatment.

Fatty acid *β*‐oxidation occurs in both mitochondria and peroxisomes, with each organelle containing distinct sets of enzymes encoded by different genes and with different specific substrates [25]. The mitochondrial *β*‐oxidation pathway primarily processes C18 and shorter fatty acids (long‐, medium‐, and short‐chain fatty acids), whereas the peroxisomal pathway handles very long‐chain fatty acids (C22 or longer), branched‐chain fatty acids, bile acid intermediates, and long‐chain dicarboxylates [26]. The substantial reduction in dicarboxylates in this study is likely due in part to peroxisomal and mitochondrial *β*‐oxidation induced by seladelpar. Peroxisomes are only capable of shortening the fatty acid chain length, and the end products of peroxisomal *β*‐oxidation (acetyl‐CoA, propionyl‐CoA, and shortened acyl‐CoA) must be transported to the mitochondria for complete oxidation via the TCA cycle and oxidative phosphorylation processes [27]. The widespread decrease in various lipid classes, including dicarboxylates, saturated and unsaturated fatty acids, and ceramides, following seladelpar treatment may thus be explained by enhanced fatty acid *β*‐oxidation in both mitochondria and peroxisomes. Circulating ceramides have been shown to have negative health consequences [28–31], so the finding here that seladelpar lowers most of the MS‐identified ceramides is intriguing. Specific lipids that have been shown to associate with initiation and resolution of inflammatory events, such as 12‐HETE [32] and 14‐HDoHE/17‐HDoHE [33], were all reduced by seladelpar.

Other major classes of metabolites increased by seladelpar treatment included *N*‐acetylamines, *N*‐acetyl amino acids, and catabolites of branched‐chain amino acids. The increase in N‐acetyl amines or N‐acetyl amino acids may result from an excess of acetyl groups, as reflected in the elevated acetylcarnitine levels [34, 35]. The rise in branched‐chain amino acid catabolites may contribute to fuel switching in conditions of reduced glucose metabolism [17, 36]. Glycerol 3‐phosphate, an intermediate in the glycolytic pathway that plays a central role in energy production, was increased significantly by seladelpar and could reflect elevated fat mobilization and lipolysis.

Cholestasis in PBC is known to cause mitochondrial disturbances, leading to elevated levels of fatty acids and other lipids [11, 12, 37]. The metabolomic data from the ENHANCE study demonstrated that many of these changes can be ameliorated with seladelpar treatment. Mitochondrial disturbances in PBC may contribute to the fatigue commonly associated with the disease [13]. Nevertheless, a limitation in our current study is the ability to control and understand differences in diet and the interaction of microbial–host metabolism both in healthy volunteers and in patients with PBC. In this context, the current study offers guidance for future studies to analyze the association of metabolite changes (both host and microbial, and the interaction thereof) in PBC and in the presence of therapeutics with measures of fatigue and quality of life.

Untargeted serum metabolomics from clinical trial samples provides a large‐scale approach to identifying mechanistic, pharmacodynamic, and diagnostic biomarkers. In the current study, we used advanced LC‐MS/MS analysis and bioinformatics to identify broad classes of metabolites that can serve as markers for seladelpar activity in PBC. These findings also pave the way for future exploration of how metabolite classes, their interactions, and individual molecules contribute to changes of metabolic state induced by a therapeutic agent and how these changes might work together to ameliorate disease or its symptoms.

**Nomenclature**
ALP: alkaline phosphatase
ALT: alanine aminotransferase
AMA: antimitochondrial antibodies
AST: aspartate aminotransferase
BCAT: branched‐chain aminotransferase
BMI: body mass index
DC: dicarboxylate
ESI: electrospray ionization
FDR: false discovery rate
GGT: gamma‐glutamyl transferase
HDL: high‐density lipoprotein
LDL: low‐density lipoprotein
OOB: Out‐of‐Bag
PBC: primary biliary cholangitis
PBS: phosphate buffered saline
PPAR: peroxisome proliferator‐activated receptor
PPAR*δ*
: peroxisome proliferator‐activated receptor‐*δ*
PSC: primary sclerosing cholangitis
RP: reverse phase
TOM: topological overlap matrix
UDCA: ursodeoxycholic acid
ULN: upper limit of normal
WCNA: weighted correlation network analysis

## Author Contributions

Y.J.C., C.A.M., and J.D.J. contributed to the study concept and study design. A.S. provided technical oversight and raw data analysis for the metabolomics portion of the study. J.L. and S.M.C. conducted statistical analysis. Y.J.C. and J.D.J. were responsible for data collection and wrote the manuscript. All authors interpreted data, reviewed, and revised drafts of the manuscript.

## Funding

This study was supported by Gilead Sciences, 10.13039/100005564.

## Disclosure

All authors approved the final manuscript for submission.

## Conflicts of Interest

Yun‐Jung Choi declares former employment with CymaBay Therapeutics Inc., a Gilead Sciences Inc., company, at the time this research was conducted.

Andrew Schwab declares employment with Metabolon Inc.

James Lim declares employment with Monoceros Biosystems, LLC.

Samantha M. Carlisle declares employment with Monoceros Biosystems, LLC.

Charles A. McWherter declares former employment with CymaBay Therapeutics Inc., a Gilead Sciences Inc., company, at the time this research was conducted.

Jeffrey D. Johnson formerly consulted with CymaBay Therapeutics Inc., a Gilead Sciences Inc., company, at the time this research was conducted.

CymaBay Therapeutics, a Gilead Sciences Inc., company, funded the study.

## Data Availability

Gilead Sciences shares anonymized individual patient data upon request or as required by law or regulation with qualified external researchers based on submitted curriculum vitae and reflecting non conflict of interest. The request proposal must also include a statistician. Approval of such requests is at Gilead Science′s discretion and is dependent on the nature of the request, the merit of the research proposed, the availability of the data, and the intended use of the data. Data requests should be sent to datarequest@gilead.com.
